# Evaluating the Effectiveness of Text Messaging and Phone Call Reminders to Minimize No Show at Pediatric Outpatient Clinics in Pakistan: Protocol for a Mixed-Methods Study

**DOI:** 10.2196/resprot.9294

**Published:** 2018-04-10

**Authors:** Sana Saeed, Noureen Somani, Fatima Sharif, Abdul Momin Kazi

**Affiliations:** ^1^ Department of Paediatrics and Child Health Aga Khan University Karachi Pakistan

**Keywords:** text messaging, mobile phone, mhealth, appointments and schedules, outpatient services, pediatrics

## Abstract

**Background:**

Missing health care appointments without canceling in advance results in a *no show*, a vacant appointment slot that cannot be offered to others. No show can be reduced by reminding patients about their appointment in advance. In this regard, mobile health (mHealth) strategy is to use text messaging (short message service, SMS), which is available on all cellular phones, including cheap low-end handsets. Nonattendance for appointments in health care results in wasted resources and disturbs the planned work schedules.

**Objectives:**

The purpose of this study is to evaluate the efficacy of the current text messaging (SMS) and call-based reminder system and further explore how to improve the attendance at the pediatric outpatient clinics. The primary objectives are to (1) determine the efficacy of the current clinic appointment reminder service at pediatric outpatient clinics at Aga Khan University Hospital, (2) assess the mobile phone access and usage among caregivers visiting pediatrics consultant clinics, and (3) explore the perception and barriers of parents regarding the current clinic appointment reminder service at the pediatric outpatient clinics at Aga Khan University Hospital.

**Methods:**

The study uses a mixed-method design that consists of 3 components: (1) retrospective study (component A) which aims to determine the efficacy of text messaging (SMS) and phone call–based reminder service on patient’s clinic attendance during January to June 2017 (N=58,517); (2) quantitative (component B) in which a baseline survey will be conducted to assess the mobile phone access and usage among parents/caregivers of children visiting pediatrics consultant clinics (n=300); and (3) qualitative (component C) includes in-depth interviews and focus group discussion with parents/caregivers of children visiting the pediatric consultancy clinic and with health care providers and administrative staff. Main constructs will be to explore perceptions and barriers related to existing clinic appointment reminder service. Ethics approval has been obtained from the Ethical Review Committee, Aga Khan University, Pakistan (4770-Ped-ERC-17).

**Results:**

Results will be disseminated to pediatric quality public health and mHealth communities through scientific meetings and through publications, nationally and internationally.

**Conclusions:**

This study will provide insight regarding efficacy of using mHealth-based reminder services for patient’s appointments in low- and middle-income countries setup. The finding of this study will be used to recommend further enhanced mHealth-based solutions to improve patient appointments and decrease no show.

## Introduction

Nonattendance for appointments in health care results in wasted resources and disturbs the planned work schedules. Missing health care appointments without canceling in advance results in a “no show,” a vacant appointment slot that cannot be offered to others. In 2015, the UK Secretary of State for Health estimated that missed general practitioner and hospital appointments cost the National Health Service an estimated £912 million per year [1], and most appointments are missed due to simple reasons such as forgetfulness [2,3].

Mobile health, or mHealth, is defined as medical and public health practices supported by mobile devices. There has been a drastic increase in the usage of mobile phones with around 7 billion mobile phone subscribers globally, 89% of whom live in developing countries [4,5]. The portability of mobile phones makes this a flexible means of communication through which people can be contacted swiftly and can respond at their own convenience. The cost-effectiveness of this technology is reflected by the fact that even a substantial proportion of those living on less than US $1 per day have access to mobile phones and their SMS text messaging (short message service, SMS) [6]. A recent analysis assessing the socioeconomic impact of telecommunication in a developing country found that people living in rural areas benefit from telecommunication even more than those living in urban areas [6]. Mobile phones have thus changed the mode of communication among people worldwide and provide a great potential for engagement in care of patients with their health care provider [7]. SMS text messaging–based interventions have been quite effective in different programs, particularly in treatment adherence, nutrition programs, antenatal care attendance, and adherence for routine pediatric immunization [8]. In addition, available data show evidence for mobile phone–based SMS text messaging reminders to improve health care appointments’ attendance and reduce “no shows” [9,10].

“No shows” can be reduced by engaging frequently with the patient, and reminding them about their appointment in advance. In this regard, one such health strategy is the use of SMS text messaging and phone calls. Various studies have shown how SMS text messaging reminders are effective for health care appointment attendance in different settings. A recent randomized controlled trial investigating the impact of SMS text messaging reminders on attendance rates at outpatient clinics in a psychiatric hospital found that receiving an SMS text messaging reminder independently reduces the chance of missing the next appointment by 50% [11]. Another study in a university setting in Switzerland found that both, SMS text messages and telephonic reminders were equally useful in reducing the number of missed appointments; however, SMS text messages were found to be more cost-effective [12]. SMS text messaging reminders at weekly intervals have also proven to be an efficacious method of improving adherence to antiretroviral therapy in patients with HIV [13]. Wang et al reported clinical attendance to be 72% versus 42% in those to whom SMS text messaging reminders were sent as compared with the control group with no intervention [14]. A meta-analysis and systematic review also found that SMS text messaging reminders serve as a simple and efficient method of improving health service delivery, as well as conferring health benefits to the patients who receive the reminders [15].

In recent years, mobile phone usage has boomed in Pakistan, with 140 million mobile phone subscribers and 237.58 billion person-to-person SMS text messages generated in 2011 [16,17]. A potential limitation to the use SMS text messaging based interventions is the level of literacy. However, there have been mixed inputs related to preference of phones calls as compared with SMS text messages in populations of low literacy and resource-constrained settings [18,19]. Mobile phone text messages in local languages or local language written in English in combination with phone call can further reduce this gap [20]. While one SMS text message has a maximum allowance of 160 characters only, this limit might help keep the messages short and easy to understand for a low literacy population [21].

To improve patient’s clinic attendance and decrease “no show,” the Aga Khan University outpatient clinic sends automated two-way SMS text messages or telephonic calls as appointment reminders. The cost of the SMS text messages and the phone calls is borne by the Aga Khan University Hospital (AKUH). However, the reply cost of the SMS text message is borne by the patient, in which an SMS text message is sent to the patient in English with a reply option of Yes or No for attending the clinic appointment. In 2016, around 70,000 appointments were given for pediatrics consultancy clinic, out of which 21,150 (30.21%) appointments resulted in “no show,” despite the vigorous policies followed at the consultant clinic. Therefore, our aim is to evaluate the efficacy of the SMS text messaging and calls–based reminder system and further explore how to improve the attendance at the pediatric outpatient clinics at Aga Khan University Hospital, Karachi, Pakistan.

## Methods

### Study Setting

This study will be conducted at the pediatric consulting clinics of the Aga Khan University Hospital, Karachi, Pakistan. AKUH is a 560-bed tertiary care hospital that is accredited by the Joint Commission International (JCI) and is located in Karachi, the largest city of Pakistan with a population of over 14.9 million [22]. The Department of Pediatrics and Child health is one the biggest service providing facility across Pakistan with over 120-bed capacity. Along with general pediatrics, it provides a number of subspecialty services, including, Pediatric Neurology, Neonatology, Cardiology, Intensive care, Infectious diseases, Metabolic, Genetics, Endocrinology, Nephrology, Pediatric Surgery, and Rheumatology. The department provides extensive outpatient services in each of these specialties in the form of morning, afternoon, and evening clinics. Patient turnover last year (2016) in outpatient Pediatrics Clinics was 98,074.

### Study Design

**Figure 1:**
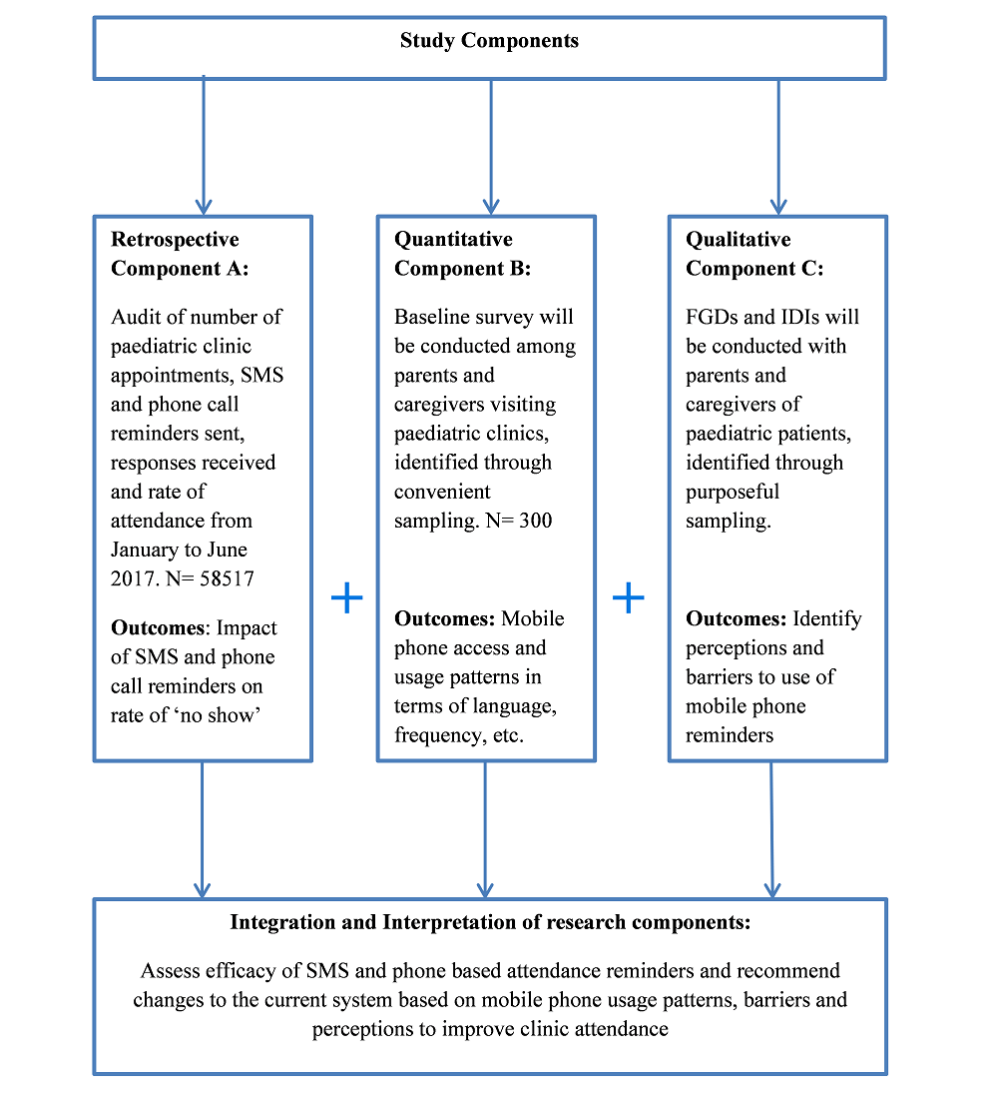
The mixed-method design of the study, which consists of three components: retrospective data, qualitative data, and quantitative data. SMS: short message service; FGD: focus group discussion; IDI: in-depth interview.

The study uses a mixed-method design which consists of 3 components (see Figure 1).

#### Retrospective Component A

A historical cohort data of pediatrics consultant clinic will be evaluated to determine the efficacy of phone-based reminders (SMS text messaging and call service) on patient’s clinic attendance during period of January to June 2017 (N=58,517). Data regarding patient appointments, SMS text messaging and phone call reminders, and actual patient attendance at the clinic will be collected. The primary objective is to evaluate whether SMS text messages and phone calls can improve patients’ appointment attendance at pediatric consulting clinic. The secondary objective is to compare the effect of SMS text messaging versus phone calls on improving clinic attendance.

The primary objective is to evaluate whether SMS text messages and phone calls can improve patients’ appointment attendance at outpatient clinic. For reporting characteristics of patients attending outpatient clinics of AKUH, continuous variables will be expressed as mean and standard deviation or median and interquartile range as appropriate. Categorical variables will be presented as proportions. Chi-square test or Fisher exact test (if expected frequency of each cell is <5) will be employed to determine whether there is a statistical difference in outpatient clinic attendance in group (1) receiving SMS text message, (2) receiving phone call, and (3) receiving both, SMS text message and phone call.

To study the association of patient-level characteristics with nonappearance at the clinic, a multivariable logistic regression will be applied to identify the predictors of patient’s attendance taking into consideration the mode of reminders (SMS text messaging, phone call, or both) and other characteristics that can be adjusted in the model. All statistical tests will be performed on Statistical Package for Social Sciences for Windows version 19 (IBM Corporation). A *P* value of .05 or less will be considered significant.

#### Quantitative Component B

A baseline survey will be conducted among parents or care givers of children visiting the pediatric consultant clinic through convenient sampling strategy.

##### Study Design, Setting and Timeline

This will be a cross-sectional, questionnaire-based study. The study will be conducted at the outpatient pediatric consulting clinics of Aga Khan University Hospital, Karachi. Data will be collected prospectively in 2 months’ time period.

##### Inclusion and Exclusion Criteria

Inclusion criteria comprised care givers visiting the Aga Khan University pediatric outpatient clinic for their children’s appointment and the ability to provide informed consent. The exclusion criteria were as follows: caregivers of patients enrolled as inpatient, visiting pediatric oncology clinics, as it is located in a different location, and visiting outpatient clinic other than pediatrics outpatient clinic.

##### Sample Population and Size

Parents and caregivers of children visiting the pediatric consulting clinic at the AKUH after giving written, informed consent to be a part of this survey will be included as the study population.

Convenient sampling will be used to identify the 300 parents and caregivers who consented to be a part of this study. A formal sample size calculation is not done. Since there are no baseline data on mobile phone coverage among caregivers visiting a pediatric outpatient clinic in Pakistan, we followed the sample size strategy of similar baseline study conducted in Kenya [23].

##### Sampling Methodology

A baseline survey will be conducted among parents and caregivers of children visiting the pediatric consultant clinics. Consecutive convenience sampling will be used to recruit patients. In addition, the caregivers while waiting for the appointment will be approached by the study staff. A prepiloted, structured questionnaire will be used for data collection. Trained study staff will approach the parents/caregivers of patients who would be waiting for their appointment at the clinic and will ask for a dedicated time for the interview. The survey will comprise questions assessing patient demographics, mobile phone access and usage patterns, as well as acceptability and feasibility of an SMS text message and phone based system for appointment reminders.

##### Data Collection

Baseline data will be collected on a smart device. Business rules, consistence check, and skips will be incorporated, and important fields will be marked as must enter.

##### Data Analysis

Descriptive statistical analysis will be used and result will be expressed in frequencies and percentages. Further univariate analysis using chi-square tests and logistic regression model will be applied for dichotomous variables, whereas Mann Whitney *U* test and linear regression will be applied for continuous variables. *P* value of less than .05 will be considered as significant.

#### Component C (Qualitative)

The perceptions and barriers of parents/caregivers and health care providers about minimizing the “no show” in pediatric outpatient clinics at Aga Khan University Hospital, Karachi, and perceptions and barriers related to SMS text message and phone call–based clinic attendance reminders will be explored.

##### Study Design, Participants, Setting, and Duration

The proposed study is qualitative (exploratory) in nature. Data will be collected through interviews. Interviews will be conducted with the parents/caregivers visiting the pediatric consulting clinics and health care staff providing the services. We will explore the perceptions of the parents/caregivers visiting the pediatrics consulting clinics and health care providers. This component of the study will be completed in a period of 2 months that includes data collection, data analysis, and interpretation of data.

##### Inclusion and Exclusion Criteria

We will include parents/caregivers and health care providers visiting the Pediatric Consulting Clinics and health care staff providing the services. Parents/caregivers who refused to provide consent at the time of conducting the focus group discussions (FGDs) and in-depth interviews (IDIs) or caregivers who are visiting clinics other than Pediatrics Consultant Clinics will be excluded.

##### Data Collection Process and Tools

Data will be collected through conducting FGDs and IDIs, which will be conducted with parents/caregivers visiting the Pediatric Consulting Clinics and related health care providers (including consultants, paramedic, and management staff).

Data will be collected through semistructured interview guide; the focus of these interviews will be to explore perceptions of parents/caregivers and health care providers about the barriers related to “no show” and facilitators related to mobile phone–based SMS text message reminders, phone calls reminders, and the health care appointments attendance at the consultant clinics. FGDs and IDIs will be conducted with the parents/caregivers visiting Pediatric Consulting Clinics and health care providers at the Aga Khan University Hospital stadium road campus. Purposeful sampling will be used to identify parents and caregivers. In addition, sociodemographic information including participant’s age, occupation, and education levels will be collected by using information sheet.

##### Data Analysis

All audio tapes will be transcribed by the research team as soon as possible after the data collection event. Qualitative data will be coded and analyzed through NVivo 11.4.1.1064 PRO for Windows (QSR International). Emerging themes and subthemes will be reported.

### Ethical Considerations

The ethical approval for the study has been obtained from the Aga Khan University’s Ethics Review Committee for approval before commencement of any study activities.

### Patient Data Confidentiality

All the research documents will be held confidential and only shared with individuals who are directly involved in the study. Study questionnaire data in the tablets will be transferred on daily basis to the central database and the database will reside on a central computer managed by the study data manager. Participants’ information will be given a study code, and data confidentiality will be maintained at all times. No personal identifiers will be used in any reports or publications of the study. For the qualitative component, unique ID will be allocated to the transcriptions and written notes. All the files will be kept in the password-protected computer.

## Results

Results of this study will be disseminated to the government and private hospitals/clinics’ pediatric and public health communities in Pakistan through scientific meetings and also submitted for publication in a peer-reviewed journal with an international public health audience. The results of this study may advise other organizations or countries to adopt this strategy to improve patient’s attendance and decrease “no show” through sending automated two-way SMS text message along with the telephonic calls as appointment reminders.

## Discussion

### Strengths

The retrospective part of this data will capture massive data of Consulting Clinic Pediatrics, which will facilitate in generalizing our findings. To the best of our knowledge, this is one of the biggest SMS text messaging and phone call–based clinic appointment reminder database of a pediatric unit in low- and middle-income countries setup. Furthermore, the mixed-method design will help to explore mobile phone access and usage, as well as perceptions and barriers to improve the use of mobile phone reminders for clinic attendance.

### Limitations

It is a single institution private sector study. Being a private sector hospital, we may not come across people with low socioeconomic background (cost perspective of nonresponders) having impact on mobile usage and ownership. There are chances of over- or underestimating a few barriers of nonresponding to the intervention.

## Abbreviations

AKUH: Aga Khan University Hospital
FGD: focus group discussion
IDI: in-depth interview
mHealth: mobile health
SMS: short message service
